# Case Report: Concomitant Massive Cerebral Venous Thrombosis and Internal Iliac Vein Thrombosis Related to Paucisymptomatic COVID-19 Infection

**DOI:** 10.3389/fneur.2021.622130

**Published:** 2021-02-10

**Authors:** Simone Beretta, Fulvio Da Re, Valentina Francioni, Paolo Remida, Benedetta Storti, Lorenzo Fumagalli, Maria Luisa Piatti, Patrizia Santoro, Diletta Cereda, Claudia Cutellè, Fiammetta Pirro, Danilo Antonio Montisano, Francesca Beretta, Francesco Pasini, Annalisa Cavallero, Ildebrando Appollonio, Carlo Ferrarese

**Affiliations:** ^1^Department of Neurology, San Gerardo Hospital ASST Monza, Monza, Italy; ^2^School of Medicine and Surgery, University of Milano-Bicocca, Monza, Italy; ^3^NeuroMi (Milan Center for Neuroscience), Milan, Italy; ^4^Department of Neuroradiology, San Gerardo Hospital ASST Monza, Monza, Italy; ^5^Department of Microbiology and Virology, San Gerardo Hospital ASST Monza, Monza, Italy

**Keywords:** COVID-19, cerebral venous thrombosis, internal iliac vein thrombosis, anticoagulation, thromboinflammation

## Abstract

Thrombotic complications are common in COVID-19 patients, but cerebral venous system involvement, timing after infection, optimal treatment, and long-term outcome are uncertain. We report a case of massive cerebral venous thrombosis and concomitant internal iliac vein thrombosis occurring in the late phase of paucisymptomatic COVID-19 infection. Mild respiratory symptoms, without fever, started 3 weeks before headache and acute neurological deficits. The patient had silent hypoxemia and typical COVID-19 associated interstitial pneumonia. Brain CT scan showed a left parietal hypodense lesion with associated sulcal subarachnoid hemorrhage. CT cerebral venography showed a massive cerebral venous thrombosis involving the right transverse sinus, the right jugular bulb, the superior sagittal sinus, the straight sinus, the vein of Galen, and both internal cerebral veins. Abdominal CT scan showed no malignancy but revealed an asymptomatic right internal iliac vein thrombosis. Both cerebral venous thrombosis and pelvic vein thrombosis were effectively treated with unfractionated heparin started on the day of admission, then shifted to low molecular weight heparin, with a favorable clinical course. Nasopharyngel swab, repeated twice, tested negative for SARS-CoV-2. Serological tests confirmed SARS-CoV-2 infection. Our case supports active surveillance and prevention of thrombotic complications associated with COVID-19, which may affect both peripheral and cerebral venous system. Early initiation of unfractionated heparin may lead to good neurologic outcome.

## Introduction

At the time of this writing, health care systems are facing worldwide the pandemic of the coronavirus SARS-CoV-2 and its associated disease, named COVID-19. Although COVID-19 mostly affects the respiratory system, ranging from mild flu-like symptoms to severe pneumonia, coagulopathy is a common feature of the disease and it is highly associated with poor prognosis (1, 2). Here, we report the case of a concomitant massive cerebral venous thrombosis and internal iliac vein thrombosis occurring in the late phase of a paucisymptomatic COVID-19 infection.

## Case Description

A 62 years old female patient was referred to the emergency room for acute onset of confusion, dysarthria, and right limbs weakness. In the previous 3 weeks she had dry cough, fatigue, and loss of appetite, without fever. In the last few days, she complained of a headache. No abdominal or pelvic pain was reported. Her medical history was limited to arterial hypertension. Neurological examination showed a moderately agitated patient with global aphasia, right-sided neglect, and a severe hypotonic right hemiparesis (Medical Research Council power scale 1/5 at right upper limb and 0/5 at right lower limb). Arterial blood gases showed hypoxia (paO_2_ 59 mmHg breathing room air), despite she had no dyspnea. D-dimer levels were high (2,768 ng/mL) and reactive C protein was moderately elevated (19.45 mg/dL).

## Diagnostic Assessment

**Figure 1:**
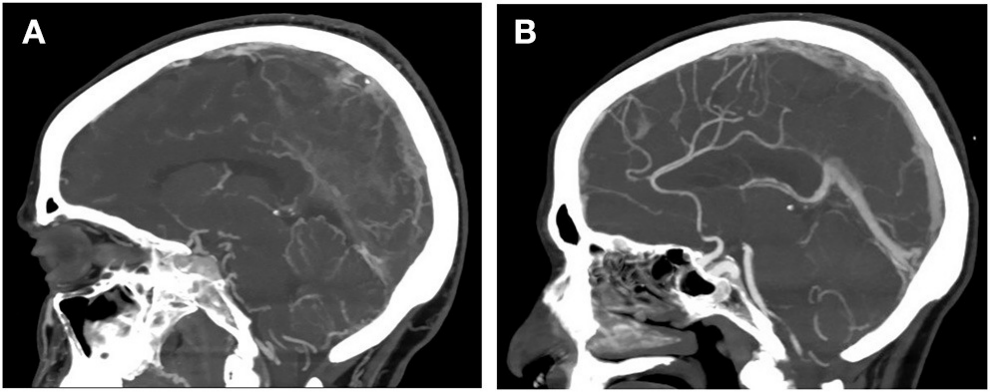
CT cerebral venography showing massive cerebral venous thrombosis associated with COVID-19 infection. **(A)** At admission. **(B)** After 3 weeks of anticoagulation therapy.

**Figure 2:**
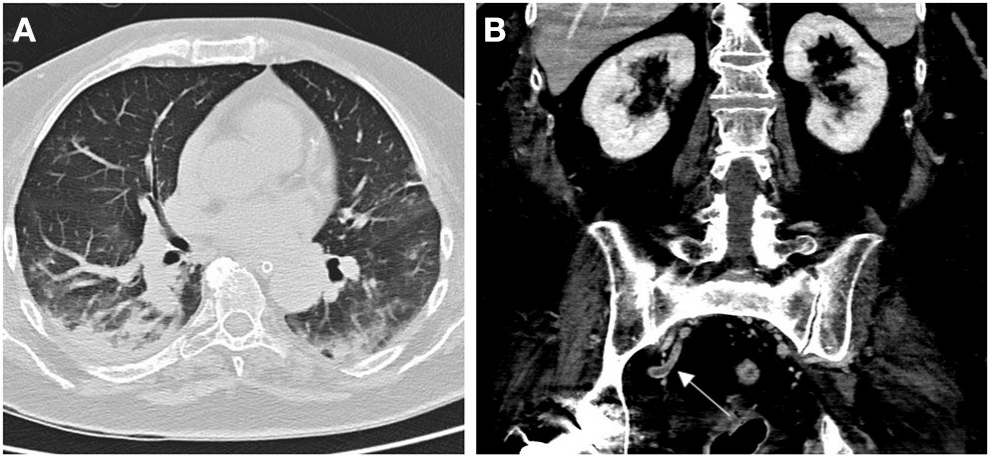
Chest CT scan showing typical COVID-19 associated bilateral pneumonia ad admission **(A)**. Contrast-enhanced abdominal CT scan showing right internal vein thrombosis (arrow; **B**).

Brain CT showed a left parietal hypodense lesion and a sulcal subarachnoid hemorrhage over the left temporal lobe. CT cerebral venography showed a massive cerebral venous thrombosis (CVT) involving the right transverse sinus, the right jugular bulb, the superior sagittal sinus, the straight sinus, the vein of Galen, and both internal cerebral veins (Figure 1A). Chest CT showed multiple bilateral ground-glass opacities and consolidations typical of COVID-19 pneumonia (Figure 2A). Infectious disease specialist was consulted and diagnosed COVID-19 on the base of typical chest CT findings, blood tests and recent history of cough and malaise. Nasopharyngel swab, repeated twice, tested negative for SARS-CoV-2. Serological test confirmed SARS-Cov-2 infection. A diagnosis of massive CVT associated with COVID-19 was made and she was admitted to the Acute Stroke Unit. On the day of admission, anticoagulation therapy was started with full-dose intravenous unfractionated heparin (UFH; 5,000 units bolus, followed by 1,000 units/hour, adjusted to aPTT), then shifted to subcutaneous enoxaparin (1 mg/kg every 12 h) after 5 days. Antibiotics (ceftriaxone and azithromycin), anti-viral therapy (lopinavir + ritonavir), and hydroxychloroquine were also started on the same day. Low dose supplemental oxygen (24–28%) was administered, with no need of further respiratory support. Contrast-enhanced abdominal and pelvic CT scan showed no malignancy but revealed a right internal iliac vein thrombosis (Figure 2B). Both CVT and iliac vein thrombosis responded well to anticoagulation therapy. Clinical course was favorable with gradual improvement in language, cognition, and right motor deficit. Follow-up brain CT and CT angiography showed reduction of venous infarct volume, resolution of subarachnoid hemorrhage and partial venous recanalization (Figure 1B). She was discharged 3 weeks later in a rehabilitation center with the advice to continue anticoagulation therapy and was shifted to warfarin. She regained functional independence and was discharged home 3 months after admission. Thrombophilia screening was negative for antithrombin deficiency, factor II and V mutations, antiphospholipid antibodies, and hyperhomocysteinemia. Protein C and protein S were not assayed, since interference with anticoagulation therapy prevented their interpretation.

## Discussion

We report a probable correlation between COVID-19 infection and concomitant thromboses of cerebral venous sinuses and internal iliac vein, the latter being a rare site for deep vein thrombosis with a significant risk for pulmonary embolism (3). Our case suggests that hypercoagulability and thromboinflammation associated with COVID-19 (4) may cause venous thrombosis at multiple unusual sites, even in the late post-viremic phase of a mild respiratory infection (when nasopharingeal swabs could give a negative result). Since the beginning of the pandemic, rare cases of CVT associated with COVID-19 have been reported (5–12). Individual patient data available are reported in Table 1. Although previous reports lack some clinical details, available data suggests that CVT is more frequent in middle-aged COVID-19 patients, presenting with altered mental status several days after the onset of a mild-to-moderate respiratory syndrome. In most of these cases, short-term outcome was in-hospital mortality (50%) or it was unreported (15%). In-hospital mortality or unreported outcome was even higher (75%) when internal cerebral veins were involved, as in our patient. Concomitant CVT and pelvic vein thromboses have not been previously reported in COVID-19 patients, probably reflecting the difficulty in diagnosis of pelvic thrombosis. However, in such cases detection of pelvic thrombosis is important to guide treatment intensity and duration. In our case, both CVT and internal iliac vein thrombosis were effectively treated with early intravenous UFH followed by enoxaparin and warfarin, leading to an excellent neurological recovery and prevention of pulmonary embolism. Although early initiation of anticoagulation therapy, immediately after diagnosis in the emergency room, partially limited hypercoagulability testing, we believe that early treatment influenced outcome and should not be delayed. Our case further supports active surveillance and prevention of hypercoagulability associated with COVID-19, even in patients with mild symptoms.

**Table 1:** Clinical features of reported individual patients with cerebral venous thrombosis related to COVID-19 infection.

**Patient (Ref.)**	**Age**	**Sex**	**Time from COVID-19 symptoms (days)**	**COVID-19 respiratory syndrome**	**Altered mental status at presentation**	**Internal cerebral veins involved**	**Elevated D-dimer**	**Initial treatment**	**Death**
1^*^	62	F	21	Moderate	Yes	Yes	Yes	UFH	No
2 (13)	38	M	10	Severe	Yes	Yes	Yes	LMWH	Yes
3 (13)	41	F	5^§^	Mild	Yes	Yes	Yes	UFH	Yes
4 (13)	23	M	7	Severe	Yes	Yes	Yes	NA	Yes
5 (14)	81	M	18	Severe	Yes	No	Yes	unAC	Yes
6 (15)	72	M	5^§^	NA	Yes	Yes	NA	unAC	Yes
7 (16)	62	F	15	Moderate	Yes	Yes	Yes	NA	NA
8 (16)	54	F	14	Moderate	No	No	Yes	NA	NA
9 (17)	44	F	14	Severe	Yes	Yes	Yes	LMWH	NA
10 (5)	29	F	7^§^	Mild	Yes	No	Yes	UFH	No
11 (6)	59	M	NA	Mild	No	No	Yes	LMWH	No
12 (7)	53	M	7	Moderate	No	No	Yes	LMWH	No
13 (8)	65	M	NA	Mild	Yes	No	NA	unAC	No
14 (9)	30s	M	4	Mild	No	No	No	Dabigatran	No
15 (9)	30s	M	NA	Mild	Yes	No	Yes	UFH	Yes
16 (10)	69	F	21	Moderate	Yes	Yes	Yes	UFH	No
17 (10)	79	F	3	Mild	Yes	No	Yes	LMWH	No
18 (11)	63	F	12	Moderate	No	No	NA	UFH	Yes
19 (12)	30	M	NA	Mild	No	No	Yes	LMWH	Yes
Median (range) or *n* (%)	54 (23–81)	52% (M:F)	10 (3–21)		81%	42%	94%		50%

*Ref., reference*.

* *Current case*.

§ *Approximation. UFH, unfractionated heparin; LMWH, low molecular weight heparin; unAC, unspecified anticoagulation therapy; n, number; NA, not available*.

## Data Availability Statement

The original contributions presented in the study are included in the article/supplementary material, further inquiries can be directed to the corresponding author.

## Ethics Statement

Ethical review and approval was not required for the study on human participants in accordance with the local legislation and institutional requirements. The patients/participants provided their written informed consent to participate in this study. Written informed consent was obtained from the individual(s) for the publication of any potentially identifiable images or data included in this article.

## Author Contributions

SB and FD had the idea for the paper. SB prepared the first draft with FD and VF. PR performed imaging acquisition and interpretation, prepared the figure, and critically reviewed the manuscript for intellectual content. SB, FD, VF, BS, LF, MP, PS, DC, CC, FP, DM, FB, and FP were involved in the clinical care of the patients and critically reviewed the manuscript for intellectual content. AC performed the virological and serological tests and critically reviewed the manuscript for intellectual content. IA and CF assisted with imaging and case interpretation and critically reviewed the manuscript for intellectual content. All authors contributed to the article and approved the submitted version.

## Conflict of Interest

The authors declare that the research was conducted in the absence of any commercial or financial relationships that could be construed as a potential conflict of interest.
